# Rectal Perforation following High-Pressure Distal Colostogram

**DOI:** 10.1055/s-0040-1709140

**Published:** 2020-05-14

**Authors:** Giulia Brisighelli, Liam Lorentz, Tanyia Pillay, Christopher J. Westgarth-Taylor

**Affiliations:** 1 Department of Paediatric Surgery, Chris Hani Baragwanath Academic Hospital, University of the Witwatersrand, Johannesburg, South Africa; 2 Department of Radiology, Chris Hani Baragwanath Academic Hospital, University of the Witwatersrand, Johannesburg, South Africa

**Keywords:** anorectal malformation, high-pressure distal colostogram, imperforate anus, rectal perforation

## Abstract

In patients with anorectal malformations and a colostomy, the high-pressure distal colostogram is the technique of choice to determine the type of malformation and thus to plan the surgical repair. Perforations associated with high-pressure distal colostograms are very rare. The aim of our study was to identify pitfalls to prevent perforation secondary to high-pressure distal colostogram. The study included two male patients and was complicated with rectal perforations secondary to high-pressure distal colostogram. Both patients had an imperforate anus without a fistula. One patient had extraperitoneal rectal perforation with progressive contrast spillage into the peritoneum and demised. The other patient developed an extraperitoneal perforation and an associated necrotizing fasciitis of his perineum and scrotum, but he recovered well after debridement. Two further cases of rectal perforation have been described in the literature. Rectal perforation, although rare, is a described life-threatening complication secondary to high-pressure distal colostogram. The cause is excessive contrast pressure. Injection of contrast should be stopped once the distal end of the colon has a convex shape. Intraperitoneal perforation may cause hypovolemic/septic shock, and patients need to be appropriately resuscitated and should undergo laparotomy. Extraperitoneal perforation requires close monitoring for possible local complications, which may necessitate early debridement.

## Introduction

Anorectal malformations (ARMs) are congenital defects of the anus and rectum that frequently involve the genitourinary tract. ARMs are a spectrum of disease that range from mild malformations with a low incidence of associated anomalies and a good outcome in terms of bowel and urinary control, to severe malformations with multiple associated anomalies and a poor urinary and fecal control outcome. Both males and females can be affected. The ARM repair can be performed primarily or in a three-stage procedure comprising a colostomy at birth, anorectal reconstruction, and stoma closure. For male patients born with an imperforate anus, the safest approach is to open a divided colostomy at birth. Prior to definitive repair, a high-pressure distal colostogram is the technique of choice to determine the type of malformation and plan definitive surgery. 1 2 3 When performing the high-pressure distal colostogram, sufficient pressure is required to distend the distal rectum adequately and to identify a possible recto urinary fistula. Inadequate distension of the distal pouch may give the false impression of an ARM without a fistula, providing insufficient or misleading information for the surgeon. 1 Conversely, excessive pressure during the colostogram can cause rectal perforation. 3 Presently, anecdotal cases of this complication have been reported, all of which were intraperitoneal perforations. 3 The aim of our study is to present two cases of rectal perforation secondary to high-pressure distal colostogram treated at our institution and to perform a literature review to identify pitfalls to prevent this serious complication.

## Case Reports

### Case1

**Fig. 1:**
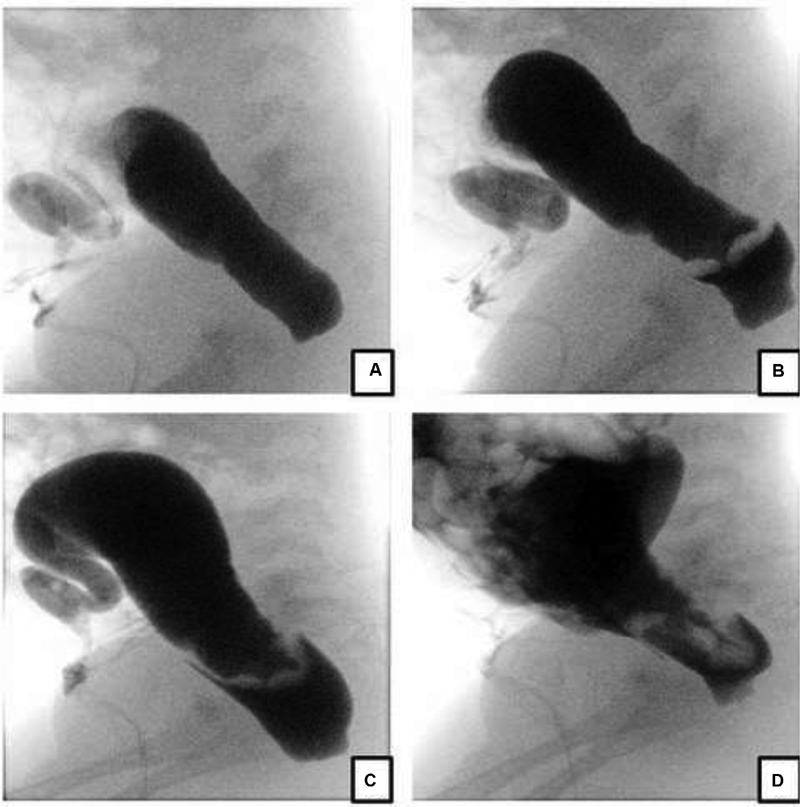
High-pressure distal colostogram lateral views (case 1). ( **a** ) Opacification of distal colon with convexity of distal rectal pouch. No rectourinary fistula is demonstrated. ( **b** ) Rectal pouch perforation with extraperitoneal contrast extravasation. ( **c** ) Progression of the extraperitoneal contrast extravasation. ( **d** ) progression of the contrast extravasation, extending into the peritoneum.

**Fig. 2:**
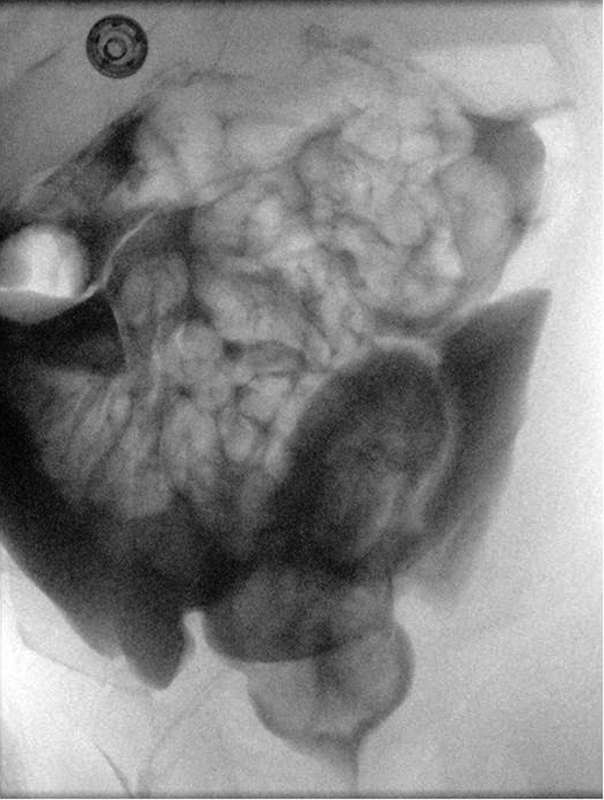
Case 1. High-pressure distal colostogram anteroposterior view showing the massive intraperitoneal extravasation of Urografin.

A term male neonate with trisomy 21 with an appropriate weight for gestational age was referred to our institution for an imperforate anus. At birth, no meconium was noted at the tip of the penis or in the urine, and a divided descending colostomy with distal mucous fistula was fashioned on day 2 of life. Screening for associated cardiac anomalies demonstrated an atrioventricular septal defect and a small patent ductus arteriosus. At 2 months of age, a high-pressure distal colostogram with water-soluble contrast medium (Urografin 30% solution) was performed, which showed an imperforate anus without a fistula. The contrast study was complicated by a rectal perforation with initial extraperitoneal contrast leak and progressive contrast spillage into the peritoneal space ( Figs. 1 and 2 ).

Due to his stable condition, soft abdomen, and normal vital signs, a decision was made to commence antibiotics (amoxicillin with clavulanic acid 20 mg/kg intravenous every 8 hours), keep the child nil per os with intravenous fluids, and manage conservatively. Unfortunately, the child's clinical condition deteriorated and a decision was then made to perform an emergency laparotomy. However, while waiting for a postoperative intensive care unit bed, the child demised.

### Case 2

**Fig. 3:**
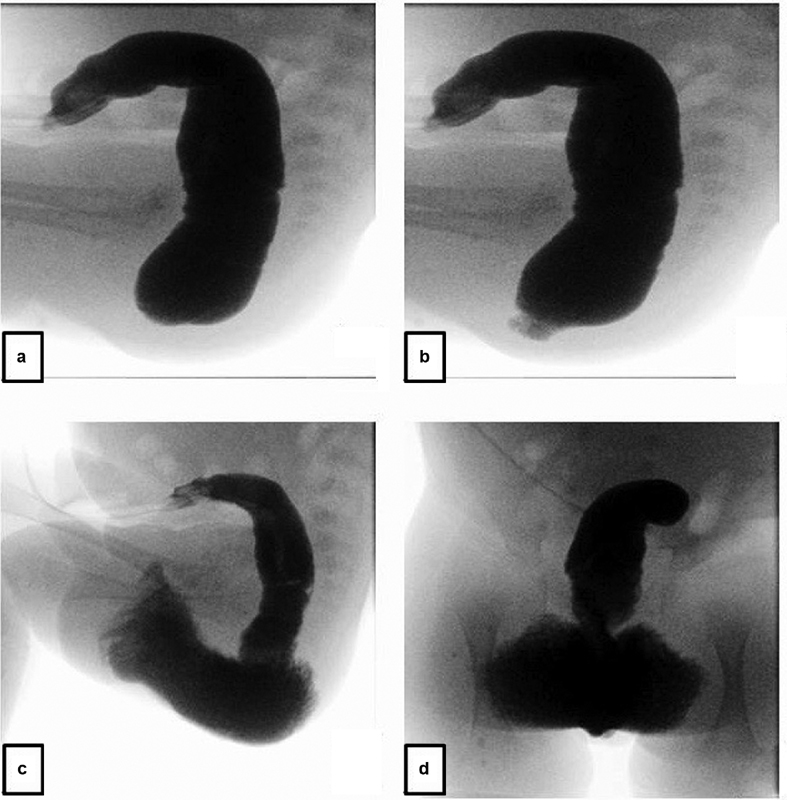
High-pressure distal colostogram (case 2). ( **a** ) Lateral view: opacification of the distal colon with convexity of distal rectal pouch. No rectourinary fistula is demonstrated. ( **b** ) Rectal pouch perforation with contrast extravasation into the perineum. ( **c** ) Lateral view: progressive perineal contrast extravasation extending into the scrotum. ( **d** ) Anteroposterior view: progressive perineal contrast extravasation extending into the scrotum.

**Fig. 4:**
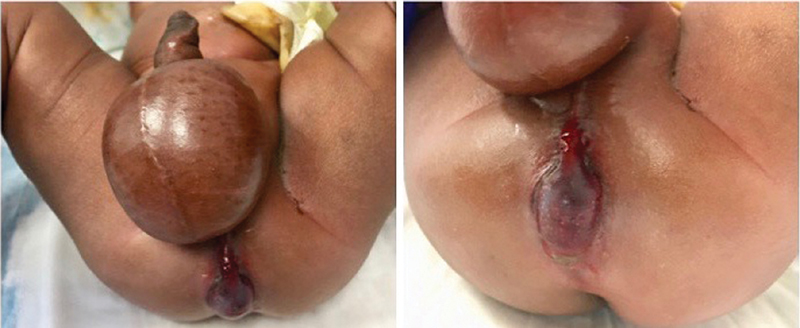
Case 2. Features of the perineum and scrotum immediately after the perforation occurred.

**Fig. 5:**
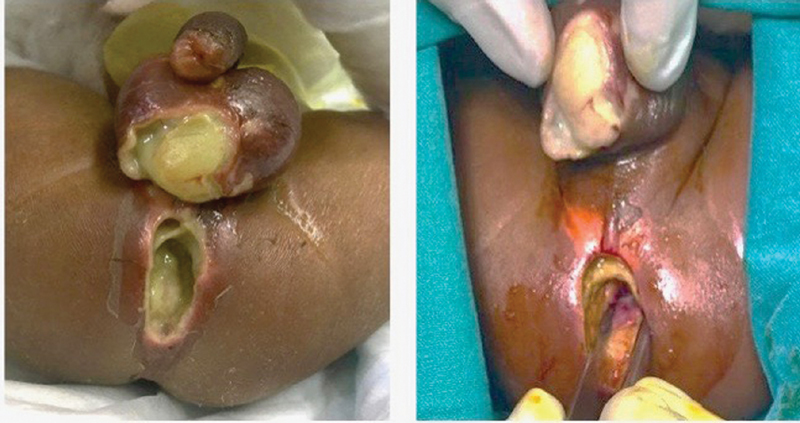
Case 2. Features of the perineum and scrotum 24 hours after the perforation occurred with macroscopic necrotizing fasciitis.

**Fig. 6:**
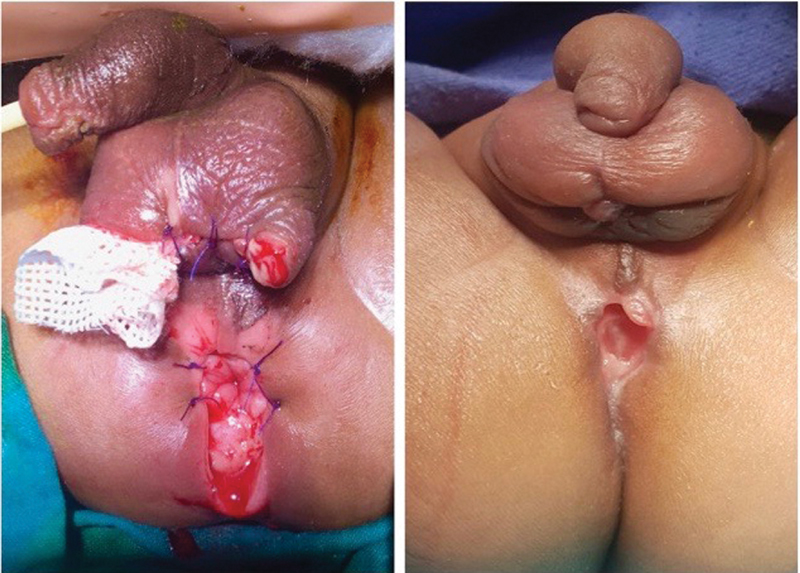
Case 2. Left: features of the perineum and scrotum 72 hours after the extraperitoneal rectal perforation (after the second debridement). Right: perineum and scrotum 6 weeks after the perforation.

A term male neonate with a birth weight of 2.7 kg and an imperforate anus was referred to our institution following the formation of a divided descending colostomy with distal mucous fistula. The child had no associated congenital anomalies. At 2 months of age, the child underwent a high-pressure distal colostogram using a water-soluble contrast medium, which showed an imperforate anus without a fistula. The contrast study was complicated by distal rectal pouch perforation and extravasation of contrast into the perineum and scrotum ( Fig. 3 ). The child was immediately admitted for observation and kept nil per os. Intravenous antibiotics were initiated and conservative management was attempted. As a result of worsening perineal inflammation and skin changes in keeping with necrotizing fasciitis, the child was taken to the operating room for debridement. Necrotizing fasciitis was macroscopically confirmed, and an extensive scrotal and perineal debridement was performed ( Fig. 4 ). Two days later, a second debridement was performed ( Fig. 5 ). Six weeks following the rectal perforation, the child underwent an examination under anesthesia, which indicated scrotal wound healing and a rectoperineal fistula anterior to the muscle complex that could fit a 4-mm Hegar dilator ( Fig. 6 ). A posterior sagittal anorectoplasty was planned at 3 months following rectal perforation to ensure adequate perineal healing.

## Discussion

The high-pressure distal colostogram was first described by Cremin et al in 1972. 4 In patients born with ARMs with a distal mucous fistula and planned for colostomy, information should be provided regarding the distance between the most distal part of the rectum and the radiopaque marker, the length of the defunctionalized colon, and the presence of a fistula between the rectum and the urinary tract. 1 Although different imaging techniques such as perineal ultrasound, pelvic magnetic resonance imaging (MRI), and voiding cystourethrogram have been described, the high-pressure distal colostogram is still considered the most important, valuable, and accurate diagnostic test to determine the position of the rectum and the presence of a fistula in patients with ARMs. 3 4 5 6 7

To perform an adequate study, a radiopaque marker (Barium paste or ball bearing) is positioned on the anal dimple, and a size 8 Foley catheter is introduced in the mucous fistula for approximately 5 cm before the catheter balloon is inflated with 2 to 3 mL of air or water. Traction is then applied on the catheter so that the balloon serves as a plug, preventing contrast leak. Using a 50-mL syringe with a catheter tip, water-soluble contrast is then injected through the Foley catheter into the rectum. The pressure is controlled by hand. With the child in a supine position, under fluoroscopic imaging, the injection commences and continues until the contrast opacifies the most distal part of the rectum. The child is then turned into a lateral position, and fluoroscopic images are taken paying attention to include the sacrum, the coccyx, the marker of the anal dimple, and the entire lower pelvis. The contrast will fill the distal rectum and stop at the pubococcygeal line. This is a horizontal line located at the pubococcygeal level and is given by the resting pelvic floor muscle tone. 8 At that point, the radiologist must exert enough hydrostatic pressure to overcome the muscle tone to distend the rectum and visualize the true location of the distal pouch and the fistula site. 3 In the case of a rectobladder neck fistula, the contrast opacifies the bladder directly with minimal hydrostatic pressure. In the case of a rectoprostatic or rectobulbar fistula, greater hydrostatic pressure is required to direct the contrast material through the rectum surrounded by muscle and through a tiny orifice communicating with the urethra. The injection of contrast must continue until the bladder is visualized and the child voids. This will allow an accurate and simultaneous visualization of the distended distal pouch, the urethra, and rectourinary fistula.

Inadequate high-pressure distal colostograms do not provide sufficient information to plan the surgical repair. This may be a result of failure to acquire images in a supine or true lateral position, lack of surface landmarks in the image (marker on anal dimple, sacrum), or, frequently, inadequate distension of the defunctionalized colon (poor seal mechanism, low injection pressure). 3 Unfortunately, there is no instrument to measure the contrast pressure in real time, and that is why the study must be performed under fluoroscopy, and the degree of distension and the resistance during injection of contrast must be evaluated. As a general rule, contrast should be injected while keeping pressure until the blind distal end of the rectum distends and become convex, overcoming the resting pressure of the pelvic floor. 1 Once there is sufficient distension of the distal rectal pouch, further contrast administration must be continued with extreme caution to avoid bowel perforation. 1 This, however, is an extremely rare complication.

In more than 1,000 male patients with ARMs surgically corrected by Peña, only two instances of bowel perforation during the injection of contrast material 3 were documented, both of which were intraperitoneal. One of the patients was initially managed conservatively but developed severe hypovolemic shock, which required immediate resuscitation followed by laparotomy to decontaminate the peritoneal cavity and address the colonic perforation. The type of perforation and the management of the second patient are not described, and there is no information regarding the type of ARM in these two patients. 3 Presently, only two cases of rectal perforation during high-pressure colostogram have been described. 3 This case series provides two additional cases and, to our knowledge, describes the only event of extraperitoneal rectal perforation.

The challenge of creating guidelines to avoid rectal pouch perforation is evident in the setting of four known cases. In our experience, and reviewing the current literature, an imperforate anus without a fistula is the type of ARM more at risk for rectal perforation. A possible reason for this could be that continued pressure is exerted with an expectation to delineate the urinary tract when no true fistula between the rectum and urinary tract exists.

In terms of management of rectal perforation secondary to the high-pressure distal colostogram, the learning point is to avoid conservative treatment for intraperitoneal rectal perforations. Unfortunately, one of our patients demised secondary to hypovolemic/septic shock after conservative treatment had been attempted. The patient's genetic and cardiac condition may have confounded resuscitation attempts and the surgical decision-making process. In the event of an extraperitoneal perforation, signs of necrotizing fasciitis, possibly due to extravasation of contaminated contrast and due to its osmotic properties, must be sought and promptly addressed. In conclusion, rectal perforation secondary to high-pressure distal colostogram is an extremely rare event and is more common in patients with an imperforate anus without a fistula. A method to avoid this complication during the distal colostogram is to abort the study once the rectum is distended, extends past the pubococcygeal line, and has a convex shape. Intraperitoneal rectal perforation should be aggressively managed, with surgery if necessary, to avoid potentially life-threatening complications. Extraperitoneal rectal perforation can be managed conservatively, but signs of local infection and necrotizing fasciitis should be evaluated and appropriately addressed.

## Notes

New Insights and the Importance for the Pediatric Surgeon An imperforate anus without fistula is the type of anorectal malformation at higher risk for rectal perforation during high-pressure distal colostogram. To avoid this complication, the procedure should be aborted once the rectum is distended, extends past the pubococcygeal line, and has a convex shape. Intraperitoneal perforation should be managed with resuscitation and prompt laparotomy. In the extraperitoneal perforation perineal necrotizing fascitis needs to be suspected and appropriately treated.
